# Two-dimensional boron nitride as a sulfur fixer for high performance rechargeable aluminum-sulfur batteries

**DOI:** 10.1038/s41598-019-50080-9

**Published:** 2019-09-19

**Authors:** Kaiqiang Zhang, Tae Hyung Lee, Joo Hwan Cha, Rajender S. Varma, Ji-Won Choi, Ho Won Jang, Mohammadreza Shokouhimehr

**Affiliations:** 1 Department of Materials Science and Engineering, Research Institute of Advanced Materials, Seoul National University, Seoul, 08826 Republic of Korea; 2 Electronic Materials Center, Korea Institute of Science and Technology (KIST), Seoul, 136-791 Republic of Korea; 3 Small & Medium Enterprises Support Center, Korea Institute of Science and Technology (KIST), Seoul, Republic of Korea; 4 Regional Centre of Advanced Technologies and Materials, Faculty of Science, Palacky University in Olomouc, Šlechtitelů 27, 783 71 Olomouc, Czech Republic

**Keywords:** Two-dimensional materials, Batteries

## Abstract

Aluminum-ion batteries (AIBs) are regarded as promising candidates for post-lithium-ion batteries due to their lack of flammability and electrochemical performance comparable to other metal-ion batteries. The lack of suitable cathode materials, however, has hindered the development of high-performing AIBs. Sulfur is a cost-efficient material, having distinguished electrochemical properties, and is considered an attractive cathode material for AIBs. Several pioneering reports have shown that aluminum-sulfur batteries (ASBs) exhibit superior electrochemical capacity over other cathode materials for AIBs. However, a rapid decay in the capacity is a huge barrier for their practical applications. Here, we have demonstrated systematically for the first time that the two-dimensional layered materials (e.g. MoS_2_, WS_2_, and BN) can serve as fixers of S and sulfide compounds during repeated charge/discharge processes; BN/S/C displays the highest capacity of 532 mAh g^−1^ (at a current density of 100 mA g^−1^) compared with the current state-of-the-art cathode material for AIBs. Further, we could improve the life-span of ASBs to an unprecedented 300 cycles with a high Coulombic efficiency of 94.3%; discharge plateaus at ~1.15 V vs. AlCl_4_^−^/Al was clearly observed during repeated charge/discharge cycling. We believe that this work opens up a new method for achieving high-performing ASBs.

## Introduction

Aluminum-ion batteries (AIBs) are considered one of the best potential alternatives to lithium-ion batteries, due in part to Al being one of the most common elements in the Earth’s crust, together with its high safety (can be directly inserted as an anode) and a higher reduction potential (−1.76 V versus a standard hydrogen electrode)^1^. In addition, AIBs have comparable theoretical gravimetric and volumetric capacities (2978 mAh g^−1^ and 8034 mAh cm^−3^, respectively)^2^ to other metal ion batteries^3–5^. A successful use of pyrolytic graphite as a cathode of AIBs shows amazing charge and discharge stability (~7000 repeated charge/discharge cycles) and higher discharge voltage (~2 V vs. AlCl_4_^−^/Al), although the electrochemical capacity is lower (~60 mAh g^−1^)^6^. Not surprisingly, research on AIBs is proliferating as many modified and improved cathode materials for AIBs have been reported in the scientific literature^7–10^ although a lack of suitable cathode materials for AIBs is a significant obstacle for high-energy-density AIBs. Cutting edge research shows a discharge capacity of ~300 mAh g^−1^ at a current density of 100 mA g^−1^ suggesting new insights and efforts are required for achieving high-performing AIBs.

Inexpensive and extremely high-capacity lithium-sulfur batteries have been intensively studied in the battery research communities with a focus on solving the problem of polysulfide shuttling^11–15^. Inspired by the high electrochemical capacities of lithium-sulfur batteries, a prospect of elemental sulfur as a cathode for AIBs shows an intriguing possibility for seriously enhancing energy densities. Several relevant reports on aluminum-sulfur batteries (ASBs) have shown the anticipated extremely high initial discharge capacity relative to previously documented carbon-based and other composite cathode materials^16–19^. However, one of the obstacles to high-performing ASBs is its limited lifespan. According to the report by Cohn *et al*., ASBs are believed to have an issue of dissolution of discharge products (polysulfide compounds)^16^; an appropriate fixer of S and polysulfide is thus required to address this impediment.

Two-dimensional materials have been extensively studied for various applications due to large specific surface area and exposed active sites^20–25^. The active sites on surfaces of two-dimensional materials may adsorb elemental sulfur and polysulfide compounds to preserve the electrochemical capacities of ASBs and thus improve the life-span by addressing polysulfide dissolution. To authenticate this hypothesis, it is necessary to classify two-dimensional materials into sulfides such as transition-metal dichalcogenides (MoS_2_, WS_2_, etc.) and non-sulfides such as boron nitride (BN) two-dimensional materials^26–28^. Recent studies on the use of these two-dimensional materials as electrodes of different types of batteries have shown highly improved performance; such materials deployed in Li-S batteries have been shown to inhibit the dissolution of polysulfide discharge products^29–32^. Deng *et al*. demonstrated that graphene supported BN nanosheets displayed an enhanced adsorption of polysulfide over a wide temperature range via the synergetic interaction of BN and graphene^33^. Additionally, these two-dimensional materials can be directly used as electrode materials for batteries^34–38^. Wang *et al*. synthesized the hierarchically free-standing WS_2_/carbon nanotube-reduced graphene oxide aerogel via a facile solvothermal method, which exhibited superior electrochemical properties as an anode for Li- and Na-ion batteries. This strategy apparently benefits from the synergetic effect between WS_2_ nanosheets and carbon nanotube/reduced graphene oxide scaffold networks and the three dimensional ordered porous structures^39^. Motivated by these advanced results, we demonstrate here the effect of both S-containing (MoS_2_ and WS_2_) and S-free (BN) layered materials on preserving electrochemical capacities during repeated charge/discharge cycling of ASBs. The incorporation of ball-milled BN/S/C, MoS_2_/S/C, and WS_2_/S/C as cathode materials, respectively, demonstrates a long-term stability and the highest capacity of BN/S/C among the reported cathode materials for AIBs.

## Results and Discussion

**Figure 1:**
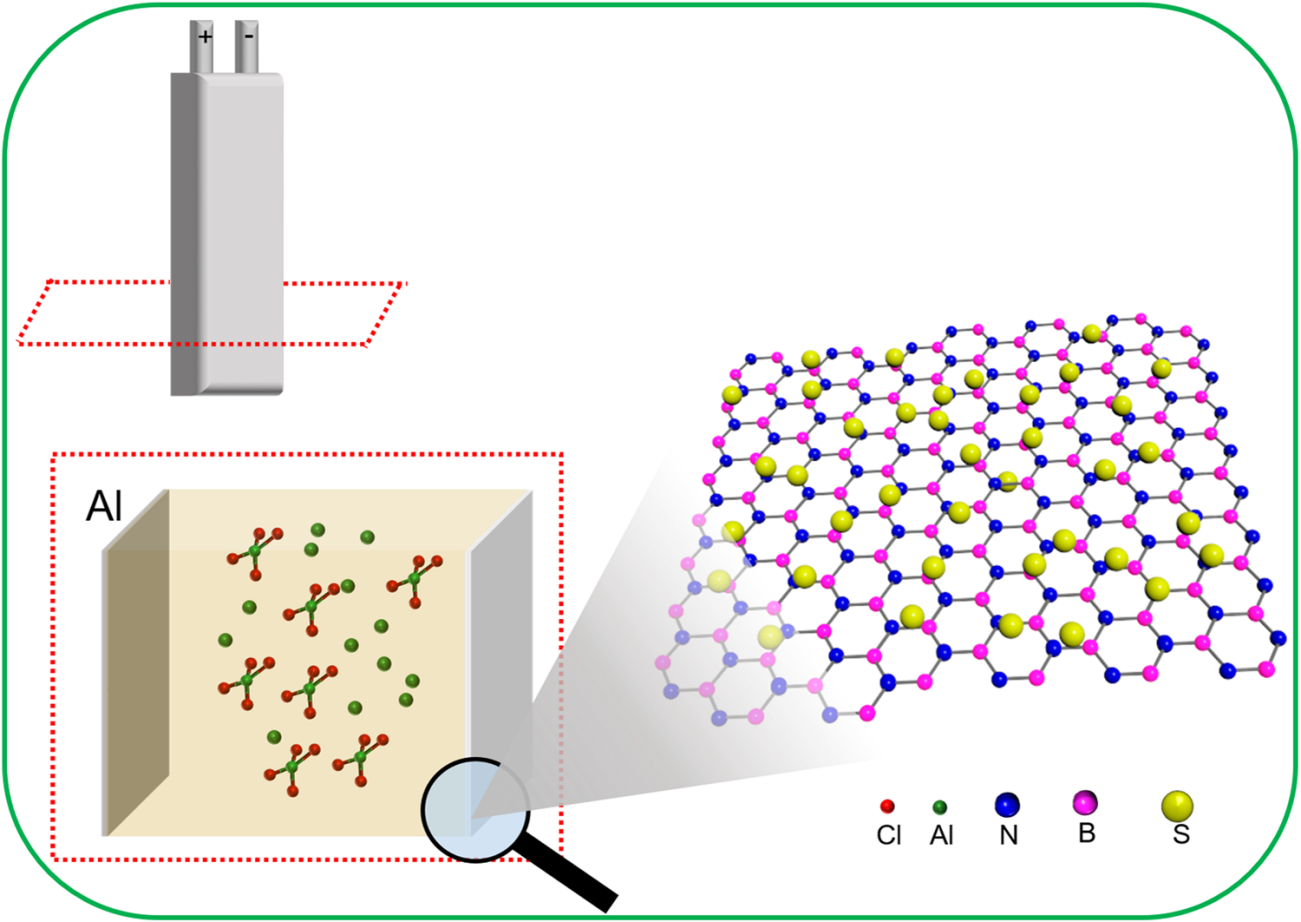
Schematic illustration of the assembled pouch cell-type ASB, with BN supported S cathode material as an example; one-layered BN here is a representative graphical illustration.

**Figure 2:**
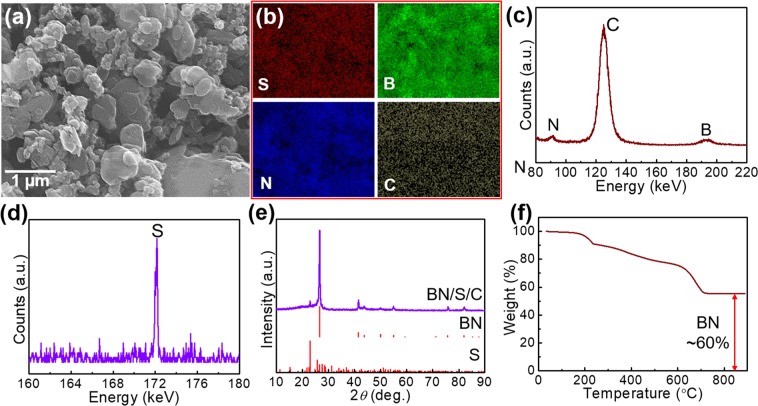
(**a**) SEM image, (**b**) EDX mapping, (**c**,**d**) EPMA spectra, (**e**) XRD spectra, and (**f**) TGA curve of the BN/S/C sample.

The ultimately assembled pouch cell is schematically shown in Fig. 1. The representative sulfur decorated BN is inserted as a cathode of an ASB. In the present report, we demonstrate three types of emerging layered materials (MoS_2_, WS_2_, and BN, Supplementary Fig. S1) as hosts to fix sulfur-active materials. After the pre-ball-milling processing, carbon- and S-nanoparticles are adsorbed on surfaces of layered materials. A uniform dispersion for them is observed in the scanning electron microscopy (SEM) and energy-dispersive X-ray (EDX) mapping results (Fig. 2a,b, and Supplementary Figs S2–S6), together with a qualitative demonstration by electron probe micro-analyzer (EPMA, Fig. 2c,d, and Supplementary Fig. S7) where corresponding elemental peaks are clearly observed. In addition, phases of S, MoS_2_, WS_2_, and BN are well preserved after the ball-milling processing (Fig. 2e and Supplementary Fig. S8). Furthermore, a transition electron microscopy (TEM) characterization for the employed components (BN, S, and C) is carried out to provide a more comprehensive study. Interplanar spacing of 0.35 nm measured in the high resolution TEM (HRTEM) image (Supplementary Fig. S9a,b) for the layered BN demonstrates a good crystallinity of commercial BN as implied in the electron diffraction pattern (Supplementary Fig. S9b inset). Furthermore, spherical C nanoparticles with a size of around 100 nm are observed in the TEM images (Supplementary Fig. S9c). Amorphous nature of the C is demonstrated in the diffraction pattern displaying circular halos (Supplementary Fig. S9d inset) which corresponds to the distorted lattices (Supplementary Fig. S9d). Besides, the spherical S particles are also demonstrated by TEM depicting a particle size of around 200 nm after ball-milling (Supplementary Fig. S9e,f). To ensure thermal stability during electrochemical measurements, thermogravimetric analysis (TGA) is performed thus verifying the consumption of only C and S at temperatures over 200 °C and without any other side reactions; slight evaporation of water molecules being the only exception when the temperature is less than 200 °C (Fig. 2f and Supplementary Fig. S10), thus confirming the adequate thermal stability at room temperature.

**Figure 3:**
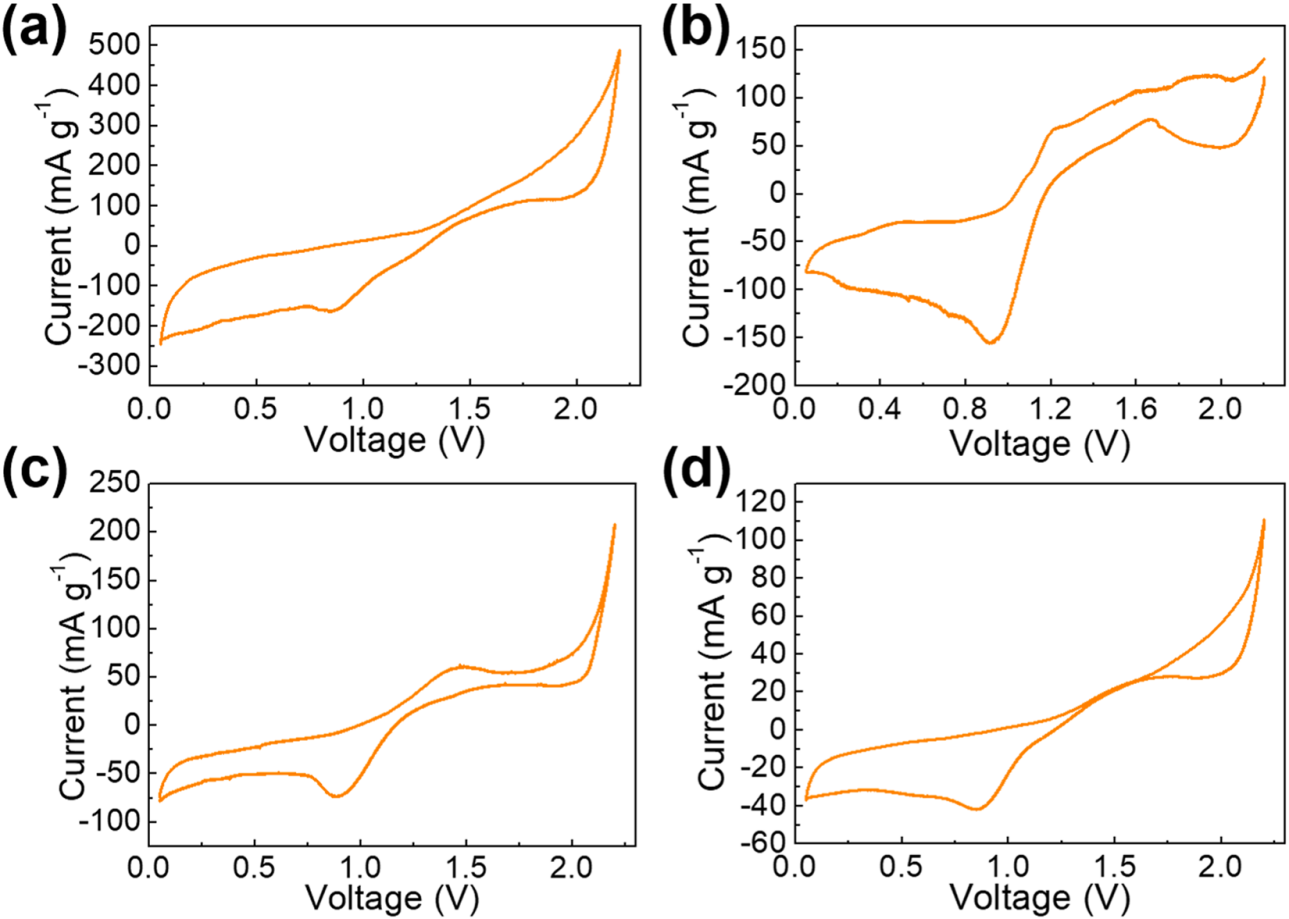
CV curves of (**a**) S/C, (**b**) WS_2_/S/C, (**c**) MoS_2_/S/C, and (**d**) BN/S/C with a scan rate of 0.5 mV s^−1^ and potential window of 0.05–2.2 V vs. AlCl_4_^−^/Al.

To determine the subsequent charge/discharge cutoff voltages, a cyclic voltammetry (CV) scan is conducted in which the S reduction peaks at ~0.8 and 2.2 V vs. AlCl_4_^−^/Al are displayed (Fig. 3a). These redox peaks are further shown in another three mixed samples (MoS_2_/S/C, WS_2_/S/C, and BN/S/C, Fig. 3b–d) at similar potentials without other obvious side-reactions. Therefore, in the subsequent electrochemical characterizations, a cutoff voltage of 0.05–2.2 V vs. AlCl_4_^−^/Al is employed.

Before measuring the electrochemical capacity of the ball-milled samples, a confirmation of capacities of the bare current collector is necessary to ensure that the capacities in the subsequent measurements intensively come from the loaded active materials rather than the current collector. As shown in Supplementary Fig. S11, the current collector donates negligibly small capacities. Subsequently, capacities of the host materials (MoS_2_, WS_2_, and BN) are measured as cathode materials of ASBs in pouch cells to ensure that these layered materials solely serve as S fixers rather than active materials.

**Figure 4:**
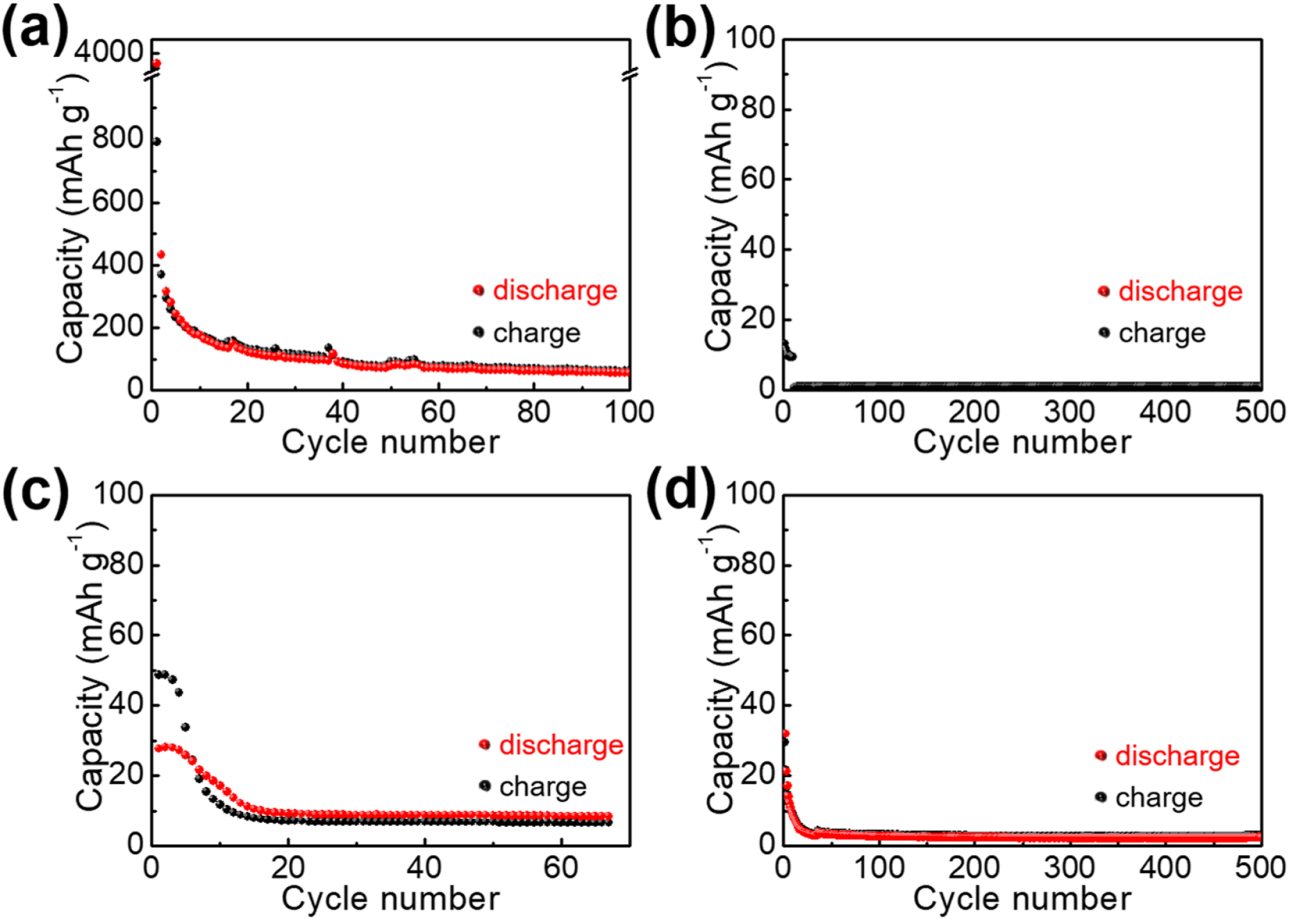
Repeated galvanostatic charge/discharge cycling measurements for (**a**) S/C, (**b**) WS_2_/C, (**c**) MoS_2_/C, and (**d**) BN/C within a potential window of 0.05–2.2 V vs. AlCl_4_^−^/Al.

**Figure 5:**
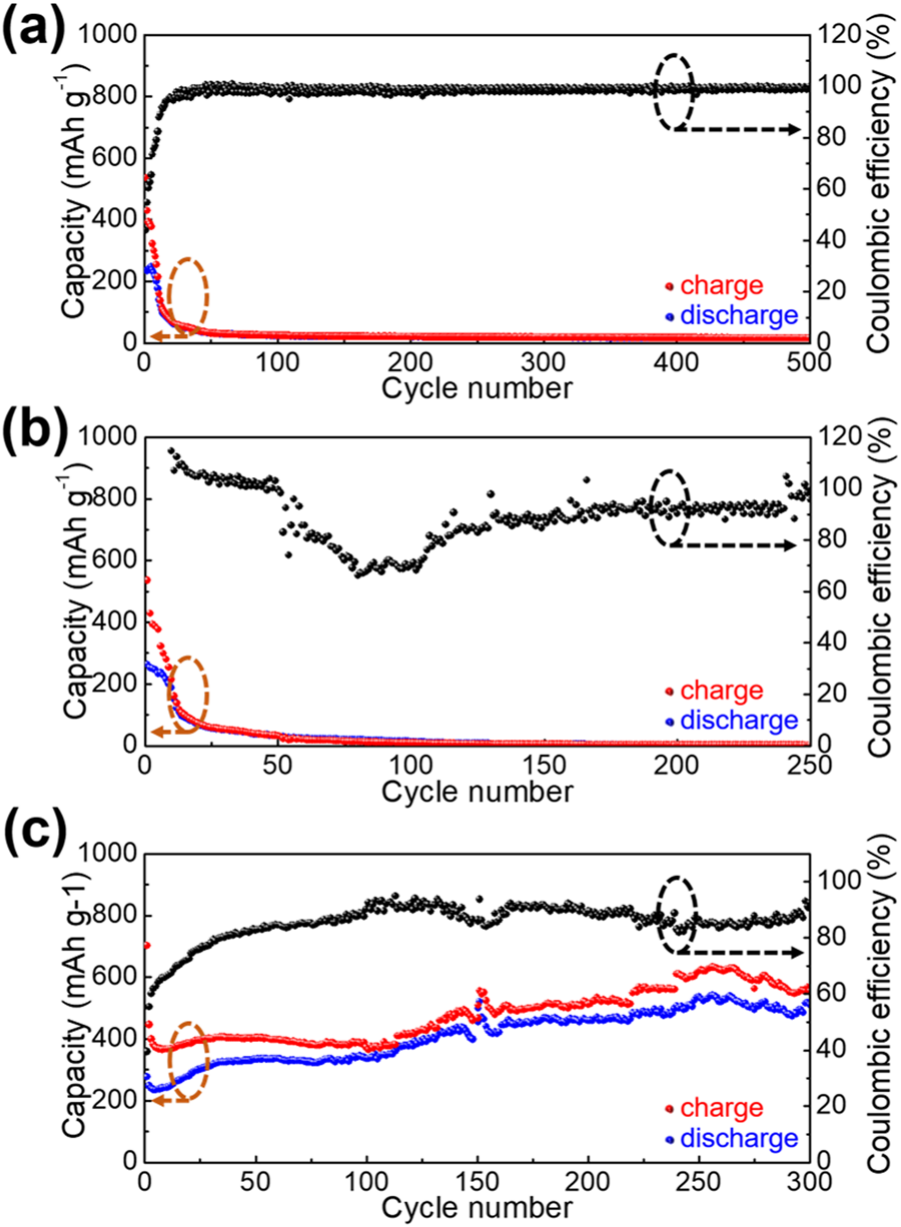
Long-term repeated charge/discharge cycling measurements for (**a**) MoS_2_/S/C, (**b**) WS_2_/S/C, and (**c**) BN/S/C at a current density of 100 mA g^−1^ within a potential window of 0.05–2.2 V vs. AlCl_4_^−^/Al.

A parallel experiment of S/C as the cathode material of an ASB is conducted for comparison. As seen in the result, S/C (Fig. 4a) exhibits a rapid decay in capacity (~50 mAh g^−1^ at the 100^th^ cycle), although it shows an initial super-high capacity (~800 mAh g^−1^). Correspondingly, the MoS_2_/C, WS_2_/C, and BN/C (Fig. 4b–d) display negligible capacities in both initial and subsequent repetitions of the charge/discharge cycles (<10 mAh g^−1^ after 10 consecutive charge/discharge cycles). This establishes that the adoption of MoS_2_, WS_2_, and BN as support materials agrees with our original proposition of layered materials strictly as S fixers rather than active materials. The effect of MoS_2_, WS_2_, and BN on an inhibition of capacity decay of S is determined by a long-term repeated charge/discharge cycling test. In the case of MoS_2_, a rapid decay from an initial 553 to ~100 mAh g^−1^ occurs after the first 20 repeated charge/discharge cycles and further decay until <50 mAh g^−1^ after the first 50 repeated charge/discharge cycles (Fig. 5a) suggests that the S and sulfide compounds may not be captured by the layered MoS_2_ via a facile ball-milling process. Similar result of negligibly small capacities for the WS_2_/S/C sample is exhibited in Fig. 5(b) where the initial discharge capacity decreases from 526 mAh g^−1^ to 54 mAh g^−1^ after the first 25 repeated charge/discharge cycles. In contrast, a completely different result is displayed for BN/S/C (Fig. 5c). A capacity of 532 mAh g^−1^ and Coulombic efficiency of 94.3% at the 300^th^ charge/discharge cycle have been obtained, although the initial capacity is similar to those of MoS_2_/S/C and WS_2_/S/C.

To the best of our knowledge, this is the highest capacity value for any graphite-based or other composite cathode materials used for AIBs. This work achieves an unprecedented long-term charge/discharge cycling stability for ASBs; additional advantages of the BN/S/C cathode material are specifically compared in Supplementary Table S1. We further demonstrate the electrochemical performance for BN/S/C mixture with enhanced S ratios. As a result, decreased capacities are obtained for BN/S/C with ratios of 5/2/2 and 4/3/2 than that with a ratio of 6/1/2 (Supplementary Fig. S12). Typically, a discharge capacity of around 100 mAh g^−1^ is retained at the 100^th^ cycle for BN/S/C with a mixing ratio of 5/2/2 which is much less than the initial high capacities suggesting the depressed capability of BN for preserving capacities of S (Supplementary Fig. S12a). This can be further demonstrated in the BN/S/C with higher S mixing ratio of 4/3/2 where the capacities quickly decay after the initial 10 cycles (Supplementary Fig. S12b).

**Figure 6:**
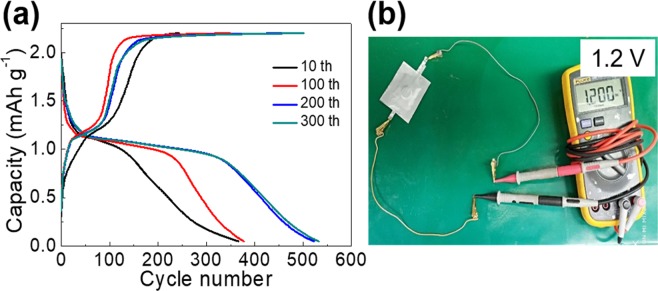
(**a**) Voltage profiles of BN/S/C at diverse charge/discharge cycles corresponding Fig. 5c. (**b**) An assembled Al//[EMIM]Cl/AlCl_3_//BN/S/C pouch cell with an open circuit voltage of 1.2 V vs. AlCl_4_^−^/Al.

In another striking result, a clear discharge plateau (~1.15 V vs. AlCl_4_^−^/Al) is shown in each charge/discharge cycle (Fig. 6a) which is quite different from other discharge plateau-free composite cathode materials for AIBs reported in scientific literature^2,7,40,41^. The high-performing ASB shows an open circuit voltage of 1.2 V vs. AlCl_4_^−^/Al (Fig. 6b). Notably, the low-cost BN/S/C, prepared by a facile ball-milling process, displaying such high performance is quite attractive for practical applications, although subsequent studies on other properties such as rate performance are warranted.

**Figure 7:**
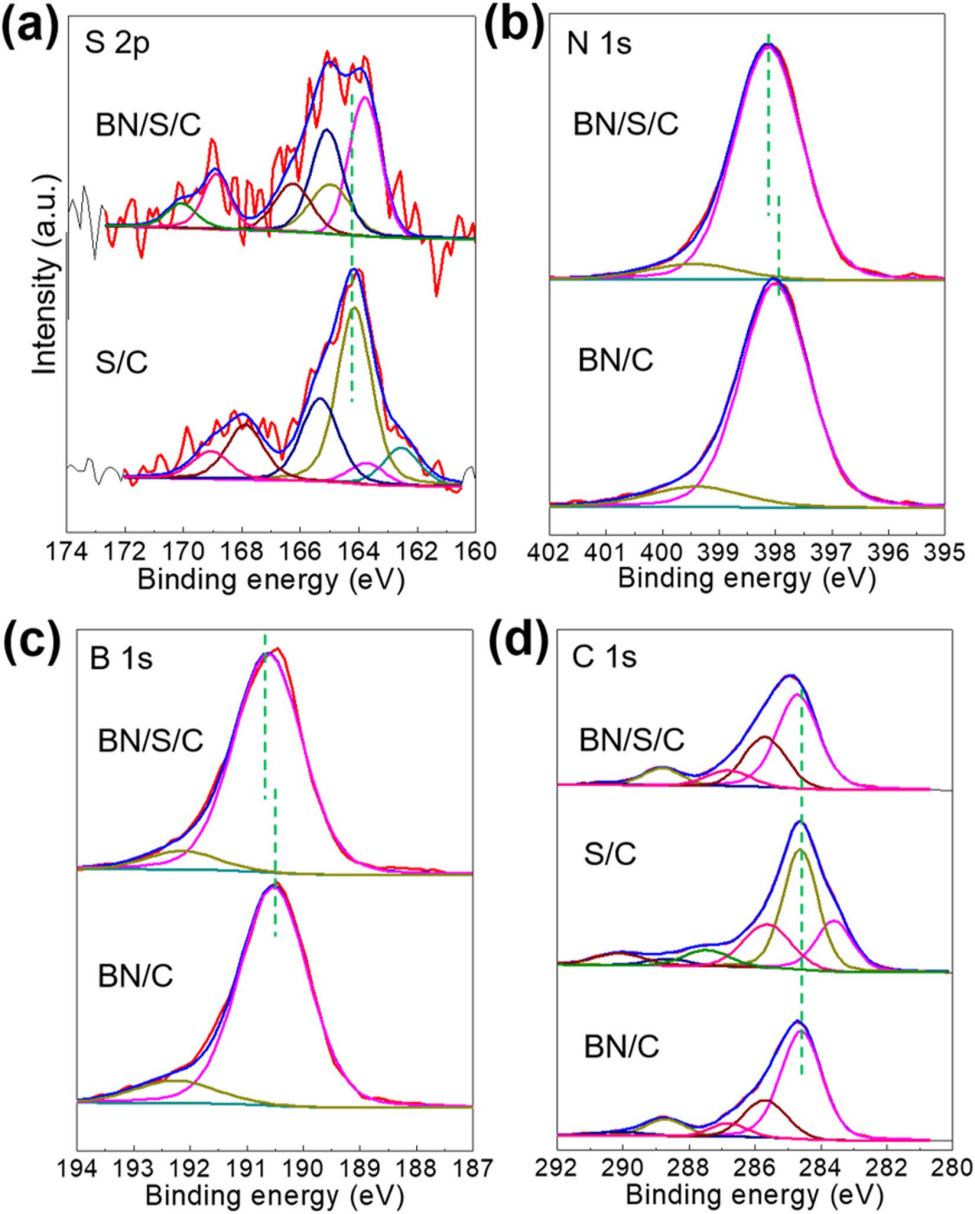
Deconvoluted XPS spectra (**a**) S 2p, (**b**) N 1s, (**c**) B 1s, and (**d**) C 1s of BN/S/C.

**Figure 8:**
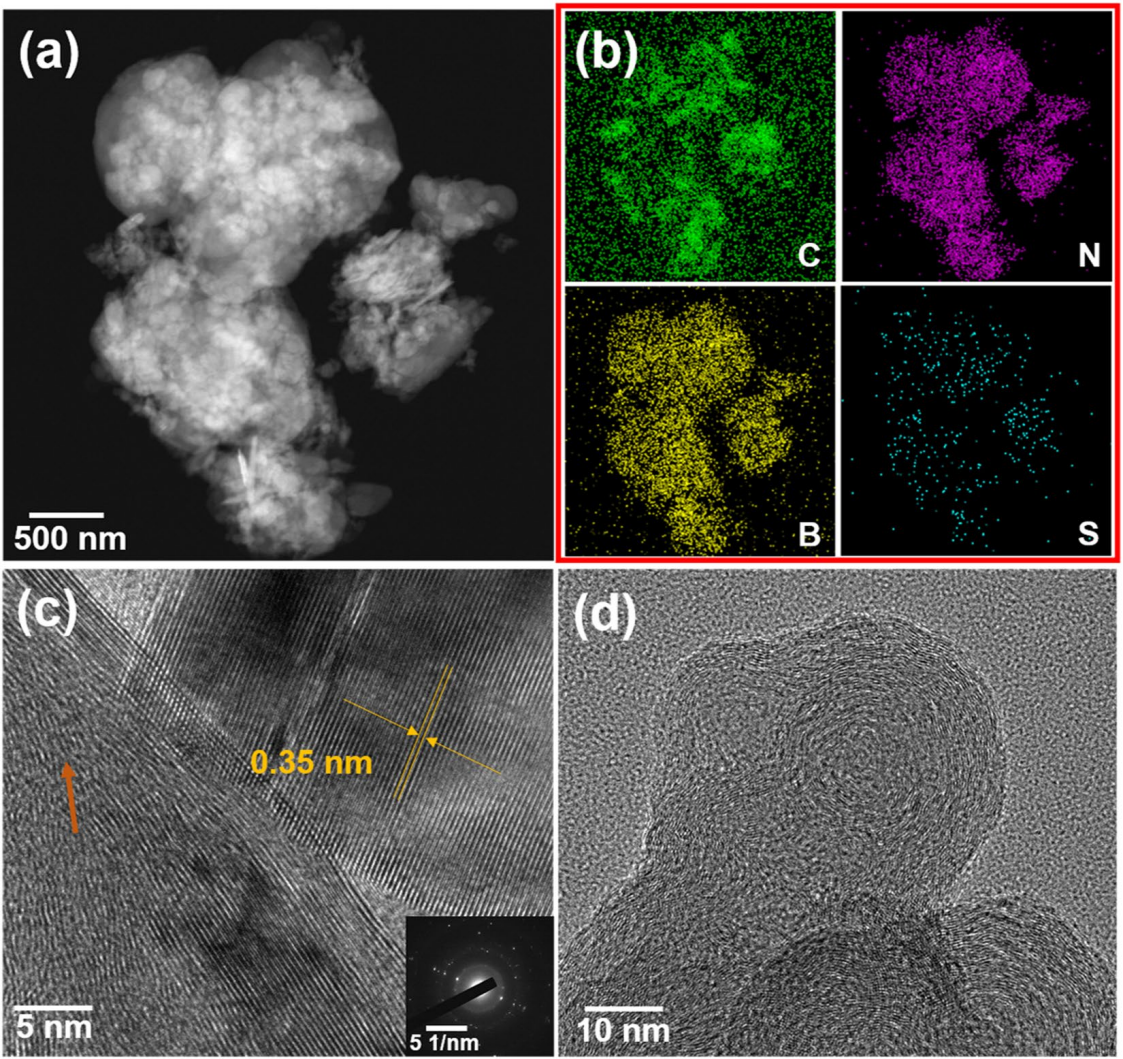
(**a**) TEM image, (**b**) EDX mapping, (**c**) HRTEM image of BN, and (**d**) HRTEM image of C in the BN/S/C sample.

If the underlying mechanism for this well-preserved capacity is consistent with our previous assumption, there should be certain variations in the bonding nature to be reflected in X-ray photoelectron spectroscopy (XPS) results. To test this hypothesis, we performed XPS analysis for the representative MoS_2_/S/C, where no obvious peak shift is revealed for any of the constituent elements (C, Mo, S, and O), indicating the bonding-free feature between S and MoS_2_ (Supplementary Figs S13 and S14). However, as we expected, the deconvoluted S 2s peaks of BN/S/C clearly shows a shift towards lower binding energy after ball-milling (Fig. 7a), demonstrating the electron adsorption from another component (C or BN). Furthermore, peaks of deconvoluted B 1s and N 1s of the BN/S/C sample shift toward higher binding energy than that of the BN/C sample (Fig. 7b,c) verifying the electron loss of N and B elements. To rule out other possibilities, that the electron transfer may be related to other elements such as C and O, we further analyzed the deconvoluted C 1s and O 1s (Fig. 7d and Supplementary Fig. S15) where almost consistent peak locations are observed. Thus, we conclude that the ball-milling processing for BN/S/C promotes electron transfer from BN to S. In other words, this facile ball-milling treatment facilitates bonding between S and BN, which can fix the sulfide compounds formed during repeated charge/discharge cycling tests. The structural feature of BN/S/C can be further expounded by TEM images and elemental mapping. The constituent elements of BN/S/C are clearly detected in TEM elemental mapping (Fig. 8a,b) where, excluding the inherent B, N, and C elements, the loaded S is uniformly distributed throughout samples. The bonding between BN and S can be further inferred by the distortion of BN lattices in HRTEM (Fig. 8c) where a disordered behavior unlike the circle-like lattice of C (Fig. 8d) is depicted compared to other well-preserved BN with a lattice spacing of 0.35 nm measured in HRTEM image. A similar behavior can be also observed in other parts of this sample (Supplementary Fig. S16).

To further reveal the underlying electrochemical reaction mechanism of BN/S/C, we carried out an elemental analysis for such samples charged and discharged till 2.2 and 0.05 V vs. AlCl_4_^−^/Al, respectively. After rinsing of the disassembled electrodes, we measure the consistent elements with EDX mapping (Supplementary Figs S17 and S18). The elements are consistently distributed throughout the powders, together with Al and Cl from remaining electrolytes and sulfide compounds. Furthermore, the formed sulfide compounds can be also found in the *ex-situ* X-ray diffraction (XRD) results of BN/S/C at different charge states (Supplementary Fig. S19a–d). In which, the formed Al_2_S_3_ in discharge products is indexed at discharge states of both 0.05 and 0.7 V vs. AlCl_4_^−^/Al. Thus, the effective charge/discharge reactions of batteries, based on the above discussion and other literature^17,18^, are formulated by the following equations.

Discharge process

1 $${\rm{Anode}}\,:\,2{\rm{Al}}+14{{{\rm{AlCl}}}_{4}}^{-}\to 8{{\rm{Al}}}_{2}{{{\rm{Cl}}}_{7}}^{-}+6{{\rm{e}}}^{-}$$

2 $${\rm{Cathode}}\,:\,8{{\rm{Al}}}_{2}{{{\rm{Cl}}}_{7}}^{-}+6{{\rm{e}}}^{-}+3{\rm{S}}\to 3{{\rm{Al}}}_{2}{{\rm{S}}}_{3}+14{{{\rm{AlCl}}}_{4}}^{-}$$

Charge process

3 $${\rm{Anode}}\,:\,8{{\rm{Al}}}_{2}{{{\rm{Cl}}}_{7}}^{-}+6{{\rm{e}}}^{-}\to 2{\rm{Al}}+14{{{\rm{AlCl}}}_{4}}^{-}$$

4 $${\rm{Cathode}}\,:\,3{{\rm{Al}}}_{2}{{\rm{S}}}_{3}+14{{{\rm{AlCl}}}_{4}}^{-}\to 8{{\rm{Al}}}_{2}{{{\rm{Cl}}}_{7}}^{-}+6{{\rm{e}}}^{-}+3{\rm{S}}$$

BN in cathode materials is well preserved after a cycling test (Supplementary Fig. S19e). Furthermore, a disappearance of elemental S diffraction peak in the cathode after a cycling test (Supplementary Fig. S19e) suggests the formation of sulfide compounds as shown in Supplementary Fig. S19f, where the formed discharge product Al_2_S_3_ is well indexed^42^. These analyses further support the proposed effective electrochemical reaction mechanism (Eqs 1–4).

An electrochemical impedance of the BN/S/C is measured with electrochemical impedance spectroscopies (EIS, Supplementary Fig. S20), where a semicircle (charge transfer process) connected with an oblique line (mass transfer process) is depicted; an electrochemical impedance of ~800 ohm and an internal resistance of less than 52 ohm are demonstrated for BN/S/C.

## Conclusions

In this report, we synthesized and comprehensively studied the immobilization effect of layered MoS_2_, WS_2_, and BN to S and sulfide compounds, and established that BN is a promising fixer to protect capacities of S from decaying by bonding with S and/or sulfide compounds. As a result, the unprecedented highest capacity is achieved for BN/S/C: 532 mAh g^−1^ with a Coulombic efficiency of 94.3% and a discharge voltage plateau at ~1.15 V vs. AlCl_4_^−^/Al when charged/discharged at a current density of 100 mA g^−1^. In addition, an unparalleled long-term life-span of 300 cycles is achieved for ASBs. The BN/S/C is validated to be superior to other reported cathode materials for rechargeable ASBs.

## Methods

### Electrode synthesis

Commercially available MoS_2_ (CAS no. 1317–33–5), WS_2_ (CAS no. 12138-09-9), S (CAS no. 7704-34-9), BN (CAS no. 10043−11-5), and super P powders were directly purchased from Sigma-Aldrich. Ball-milling (using zirconia balls) was performed for MoS_2_/S/C (6:1:2, w:w:w), WS_2_/S/C (6:1:2, w:w:w), BN/S/C (6:1:2, w:w:w), MoS_2_/C (7:2, w:w), WS_2_/C (7:2, w:w), and S/C (1:2, w:w) at 1500 rpm for 2 days to mechanically grind the powders while fixing the S powders on the layered MoS_2_, WS_2_, and BN. The ball-milled powders were further manually ground with polyvinylidene fluoride (PVDF, binder) in the ratio of 9:1 (w:w) except S/C mixed with PVDF in the ratio of 3:1 (w:w).

### Characterizations

Structural study was performed using XRD (D8-Advance equipped with Cu Ka radiation at a fixed incident angle of 2°). The surface chemical properties were analyzed via XPS (PHI 5000 VersaProbe, Al Kα source, Sigma probe, VG Scientifics). The morphologies were observed via field emission-SEM (SUPRA 55VP) and TEM (Tecnai F20). Furthermore, EDX, and EPMA were employed for the analysis of constituent elements. The thermal stability was demonstrated via TGA, which was performed under air flow from room temperature to 900 °C with a temperature ramp of 5 °C min^−1^.

### Electrochemical characterization

A slurry was prepared by dispersing the mixed powders into a constantly stirred *N*-methyl-2-pyrolidinone solution. A working electrode with a mass loading of ~3 mg cm^−2^ was prepared by spreading the slurry, once sufficiently mixed, on a Pt coated OHP organic polymer film current collector, after which the electrode was dried in a vacuum oven at 60 °C overnight.

The electrochemical properties were characterized in pouch cells assembled with the well-dried electrode as a cathode and an Al metal foil (0.5 mm) as an anode. Between two electrodes, glass-fiber paper (Whatman, 1440-070) soaked with 1-ethyl-3-methylimidazolium chloride ([EMIM]Cl)/AlCl_3_ (1/1.3, molar:molar) was inserted to isolate the anode and cathode.

CV measurements were performed on an electrochemical workstation (WBCS3000, Wonatech, Korea) in a potential range of 0.05–2.2 V vs. AlCl_4_^−^/Al at a scan rate of 0.5 mV s^−1^. Galvanostatic charge/discharge cycling measurement was performed between 0.05–2.2 V vs. AlCl_4_^−^/Al at a current density of 100 mA g^−1^. All current densities and specific capacities in the present study were calculated based on the weight of elemental S active material.

EISs of cathode materials were measured with an Im6ex ZAHNER impedance measurement facility in the same pouch cells. Used frequency range was from 10 mHz to 1 MHz with a voltage amplitude of 10 mV.

### ***Ex-situ*** characterization

The samples for *ex-situ* SEM, EDX, and XRD characterizations were prepared by disassembling the pouch cells (BN/S/C) charged to 0.05 and 2.2 V vs. AlCl_4_^−^/Al, respectively, and then cleaning sufficiently with ethanol.

## Supplementary information

Two-dimensional boron nitride as a sulfur fixer for high performance rechargeable aluminum-sulfur batteries

## Data Availability

The data that support the findings of this study are available from the corresponding authors upon reasonable request.
