# Supplementary material for: Two-dimensional boron nitride as a sulfur fixer for high performance rechargeable aluminum-sulfur batteries
Source: Sci Rep. 2019 Sep 19;9:13573. doi: 10.1038/s41598-019-50080-9 (PMC6753128; doi:10.1038/s41598-019-50080-9)
Supplement: Supplementary file 1 — Two-dimensional boron nitride as a sulfur fixer for high performance rechargeable aluminum-sulfur batteries [file 41598_2019_50080_MOESM1_ESM.docx]

**Supplementary Information**

**Two-dimensional boron nitride as a sulfur fixer for high performance rechargeable aluminum-sulfur batteries**

Kaiqiang Zhang^1,2^, Tae Hyung Lee^1^, Joo Hwan Cha^3^, Rajender S. Varma^4^, Ji-Won Choi^2*^, Ho Won Jang^1*^ & Mohammadreza Shokouhimehr^1*^

^1^Department of Materials Science and Engineering, Research Institute of Advanced Materials, Seoul National University, Seoul 08826, Republic of Korea.

^2^Electronic Materials Center, Korea Institute of Science and Technology (KIST), Seoul 136-791, Republic of Korea.

^3^Small & Medium Enterprises Support Center, Korea Institute of Science and Technology (KIST), Seoul, Republic of Korea.

^4^Regional Centre of Advanced Technologies and Materials, Faculty of Science, Palacky University in Olomouc, Šlechtitelů 27, 783 71 Olomouc, Czech Republic.

* Corresponding authors

E-mail address: jwchoi@kist.re.kr (J.-W. Choi), hwjang@snu.ac.kr (H. W. Jang), mrsh2@snu.ac.kr (M. Shokouhimehr)

**
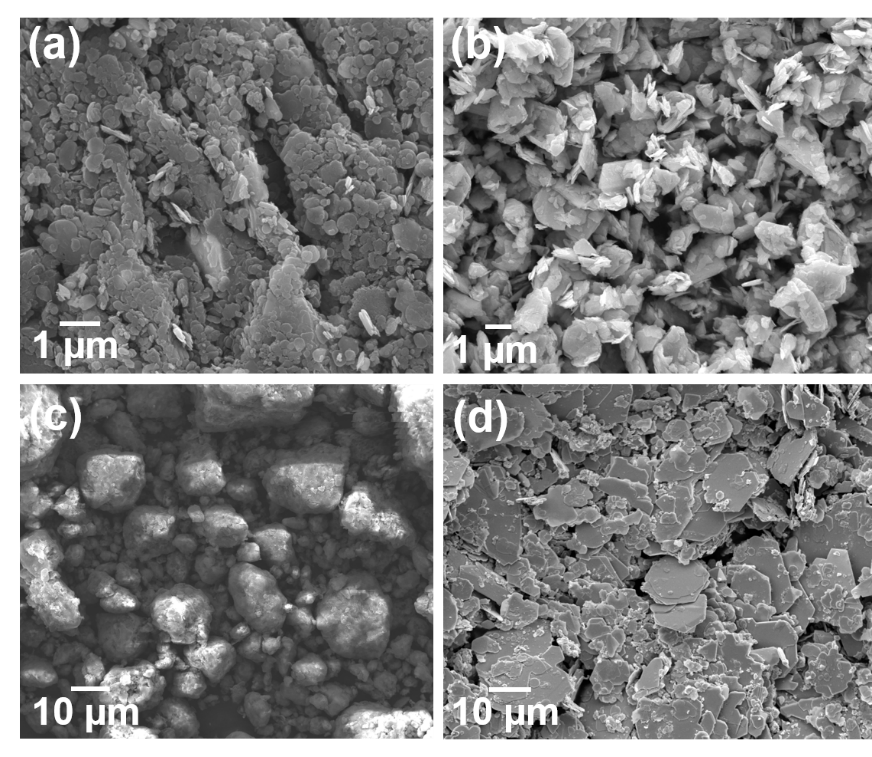
**

**Figure S1.** SEM images of the purchased raw materials: (**a**) layered BN, (**b**) layered MoS_2_, (**c**) S, and (**d**) layered WS_2_.

**
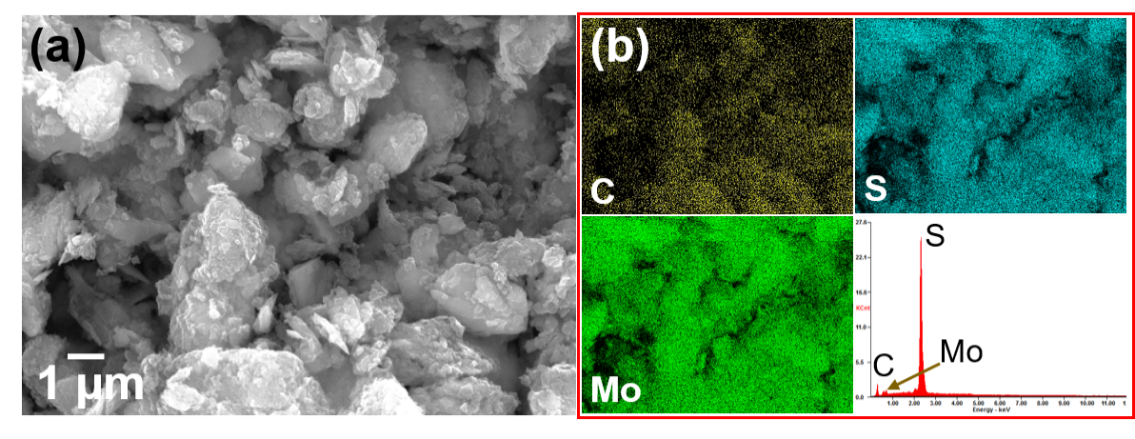
**

**Figure S2.** (**a**) SEM and (**b**) EDX mappings of the MoS_2_/S/C sample with constituent elements uniformly distributed throughout the sample.

**
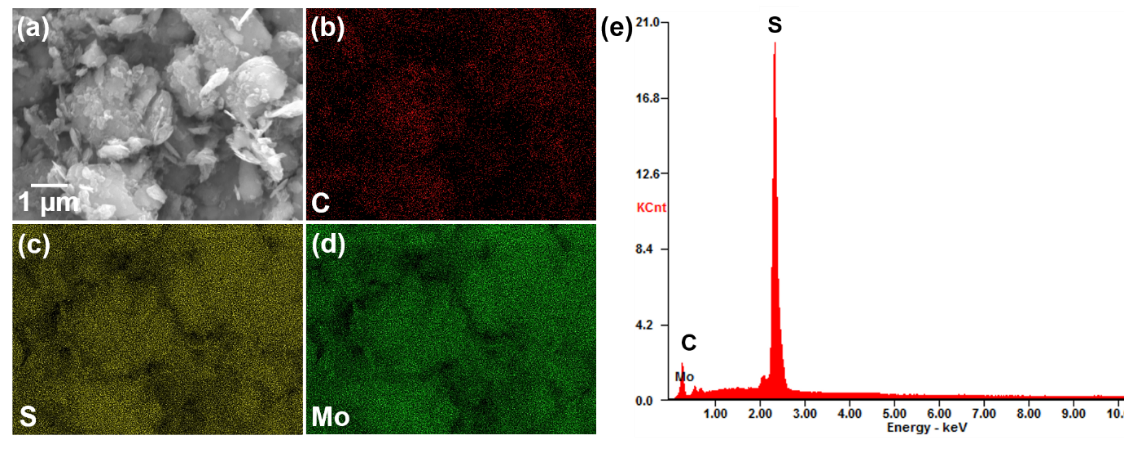
**

**Figure S3.** (**a**) SEM and (**b**-**e**) EDX mappings of the MoS_2_/C sample. C (super P) nanoparticles are well attached on surfaces of layered MoS_2_ plate via ball-milling.

**
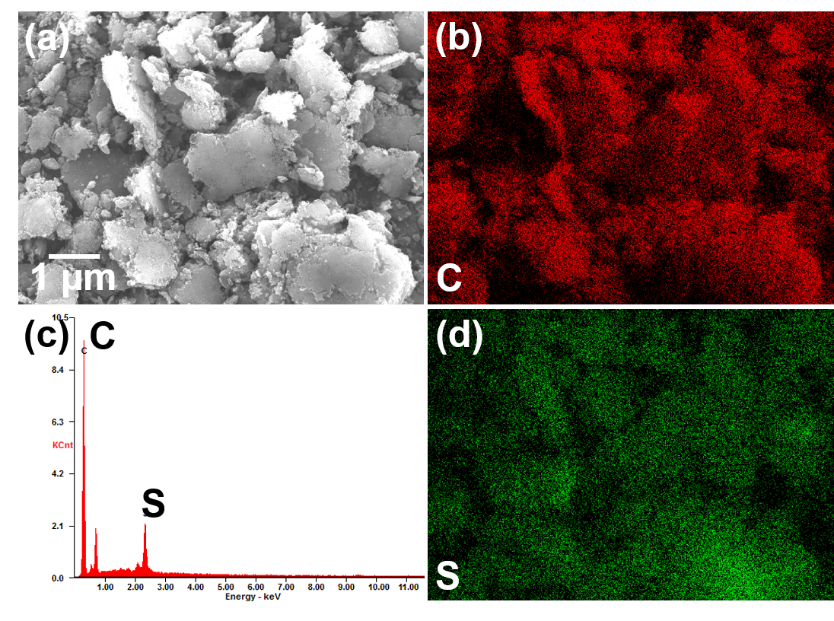
**

**Figure S4.** (**a**) SEM and (**b**-**d**) EDX mappings of the S/C sample with the S and C elements uniformly distributed throughout the sample after ball-milling.

**
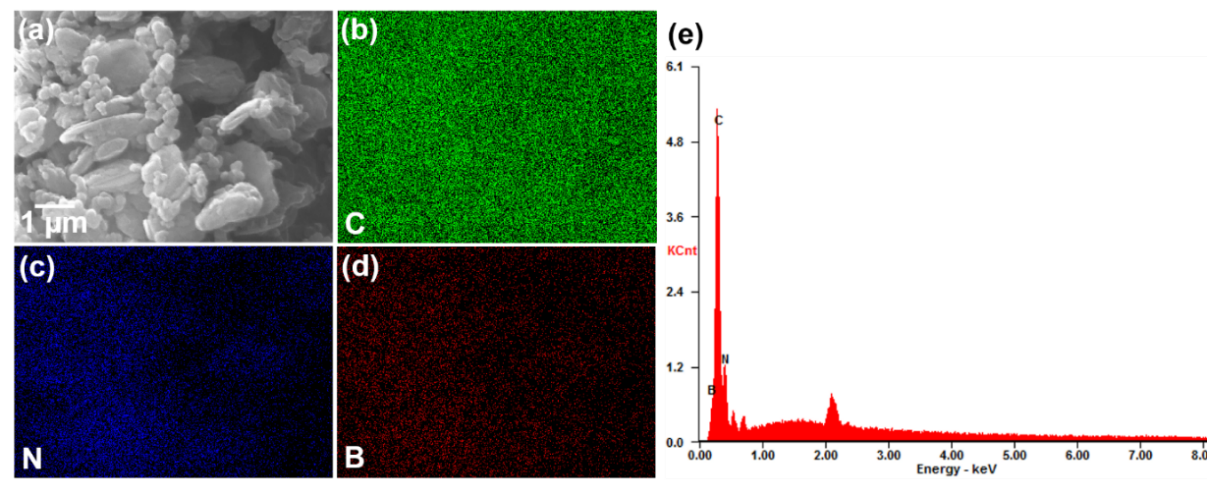
**

**Figure S5.** (**a**) SEM and (**b**-**e**) EDX mappings of the BN/C sample. C (super P) nanoparticles are well absorbed on surfaces of layered BN plates via ball-milling.

**
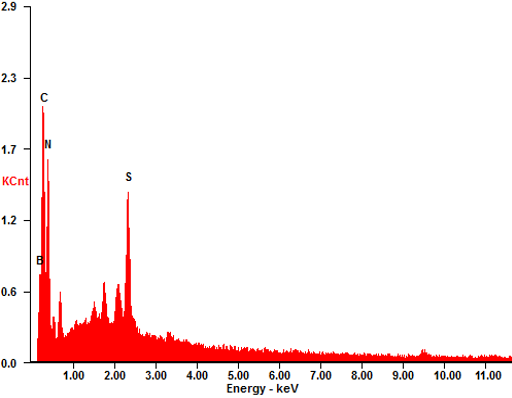
**

**Figure S6.** EDX spectra of the BN/S/C sample confirming constituent elements.

**
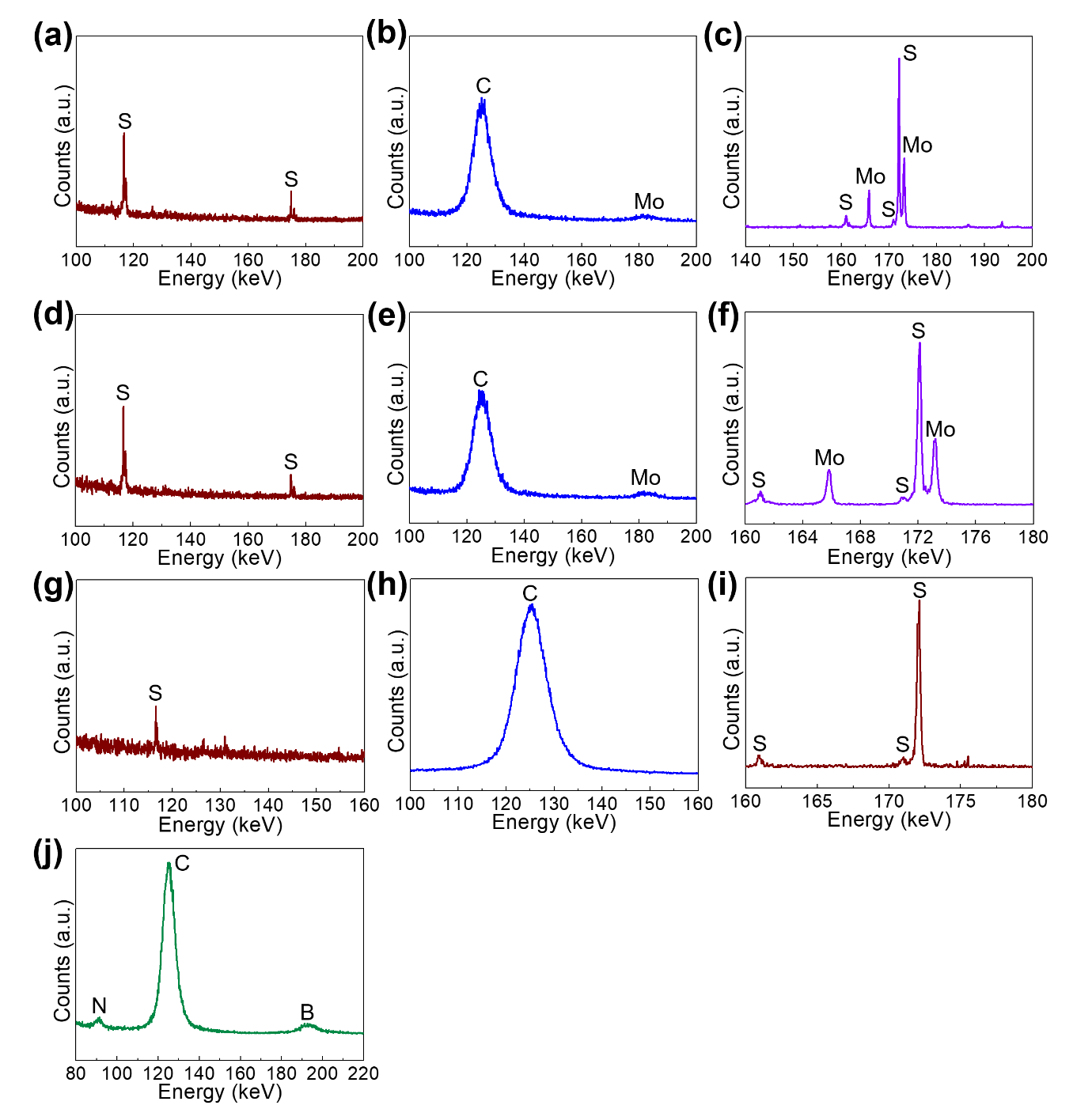
**

**Figure S7.** EPMA of the (**a**-**c**) MoS_2_/S/C, (**d**-**f**) MoS_2_/C, (**g**-**i**) S/C, and (**j**) BN/C samples further confirming the consistency of elements by screening different diffraction peaks.

**
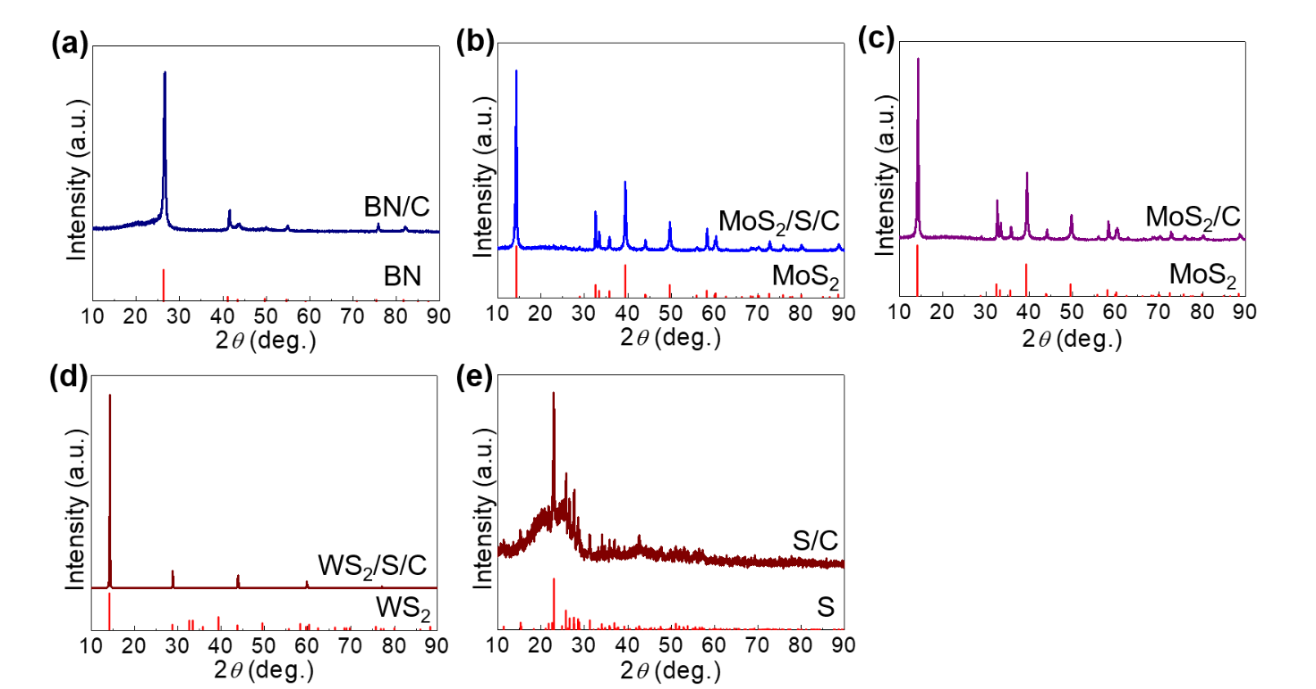
**

**Figure S8.** XRD of the (**a**) BN/C, (**b**) MoS_2_/S/C, (**c**) MoS_2_/C, (**d**) WS_2_/S/C, and (**e**) S/C samples after ball-milling. Characteristic diffraction peaks are well preserved in each sample, thus demonstrating structural integrity.

**
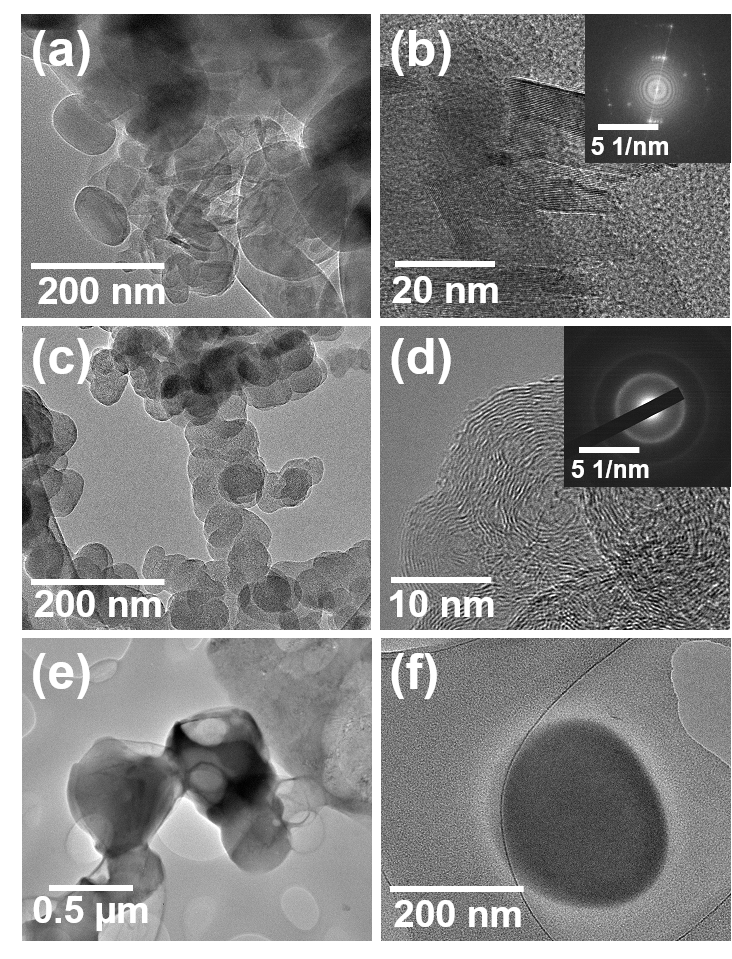
**

**Figure S9.** TEM images of bare (**a**,**b**) BN, (**c**,**d**) super P, and (**e**,**f**) S. Insets depict the electron diffraction patterns.

**
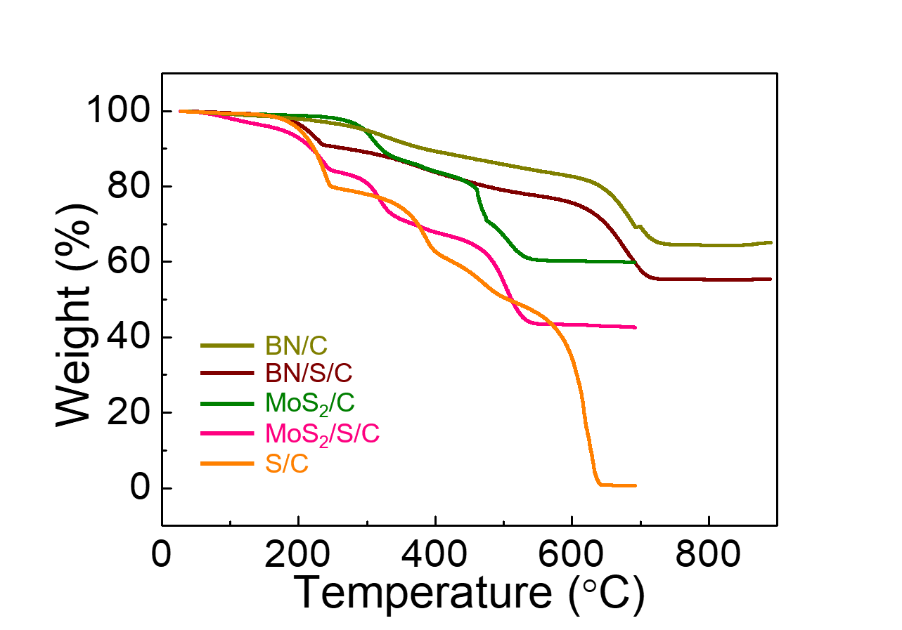
**

**Figure S10.** TGA of the MoS_2_/S/C, MoS_2_/C, S/C, BN/S/C, and BN/C samples demonstrating adequate thermal stability when used as cathode materials for ASBs.

**
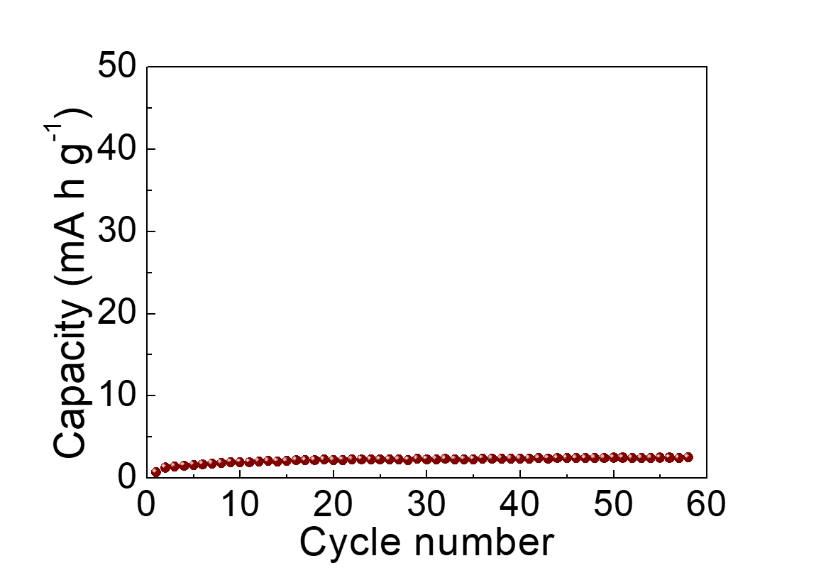
**

**Figure S11.** Capacities of a naked current collector, which is demonstrated at a current density of 50 mA g^-1^, exhibiting negligible capacities.

**
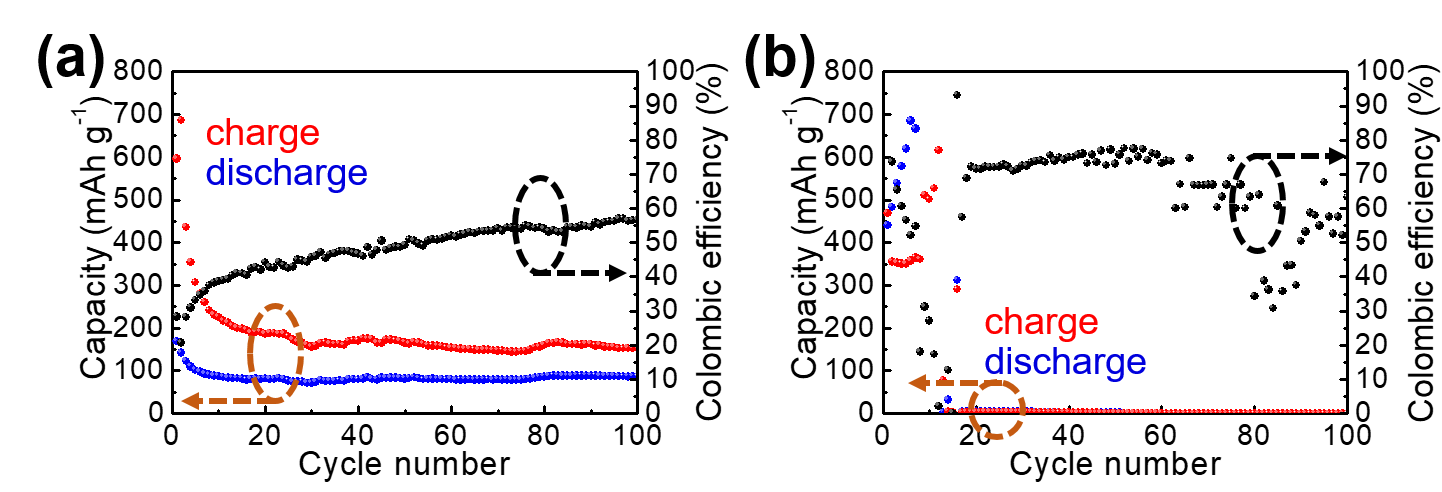
**

**Figure S12.** Repeated charge/discharge measurements for BN/S/C with BN/S ratios of (a) 5/2 and (b) 4/3.

**
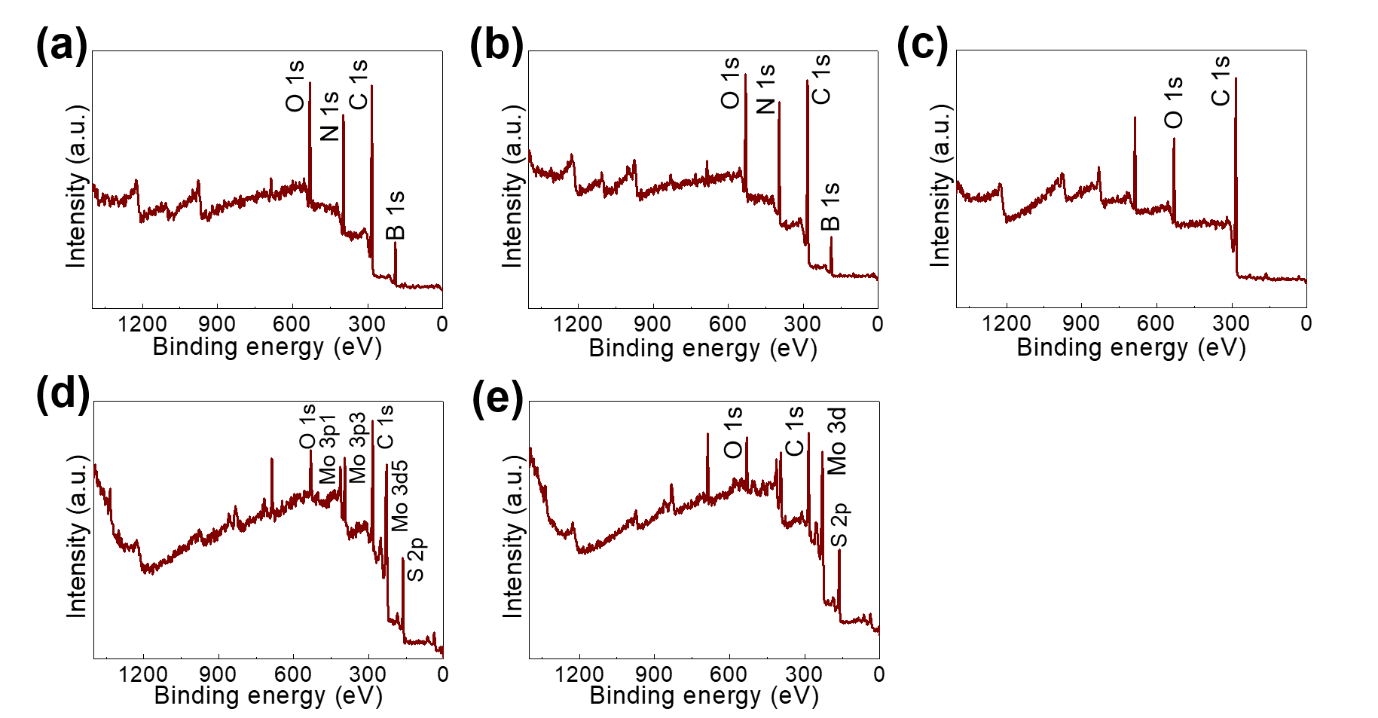
**

**Figure S13.** XPS wide survey of the (**a**) BN/S/C, (**b**) BN/C, (**c**) S/C, (**d**) MoS_2_/S/C, and (**e**) MoS_2_/C samples after ball-milling qualitatively exhibits the bonding nature.

**
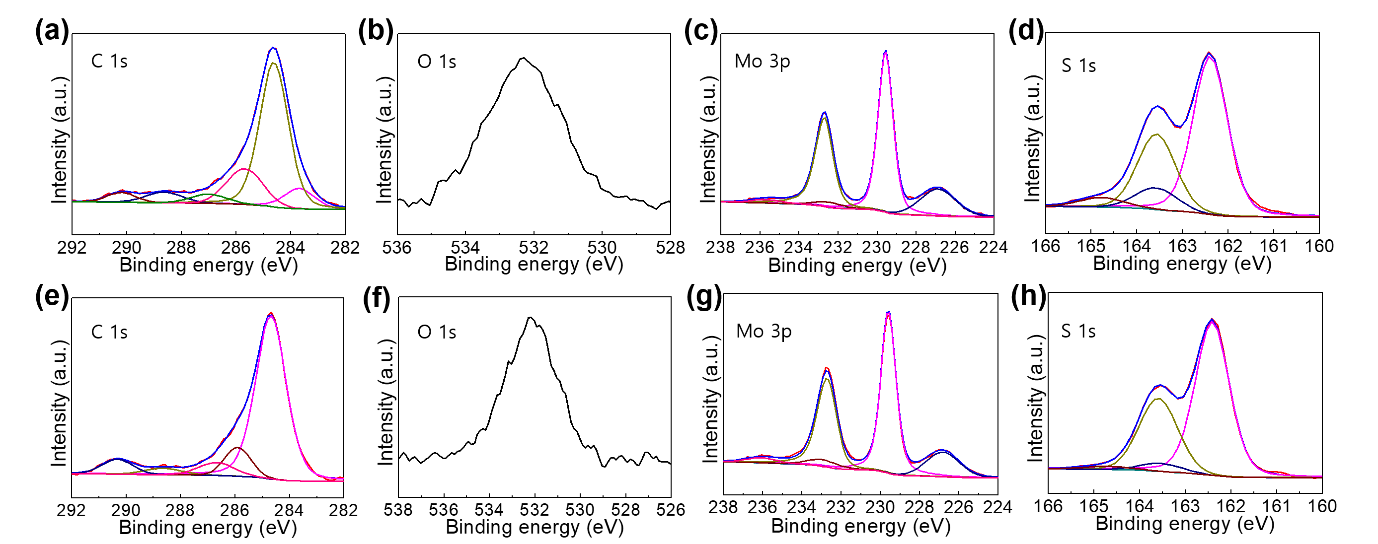
**

**Figure S14.** Deconvoluted XPS spectra of (**a**,**e**) C 1s, (**b**,**f**) O 1s, (**c**,**g**) Mo 3p, and (**d**,**h**) S 1s of the MoS_2_/S/C and MoS_2_/C samples after ball-milling displaying almost the same binding energies for each element in both samples.

**
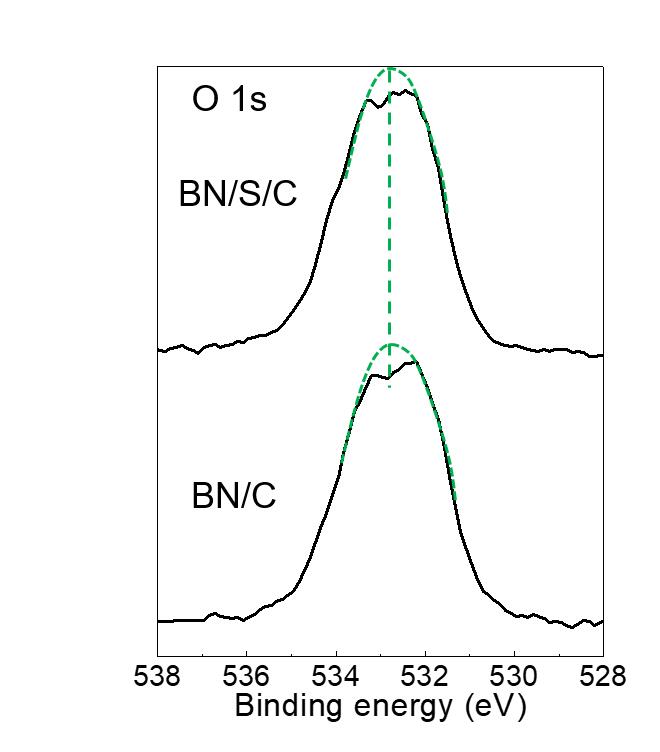
**

**Figure S15.** Deconvoluted XPS spectra of O 1s for BN/S/C (top) and BN/C (bottom) samples demonstrating the consistent binding energy of O.

**
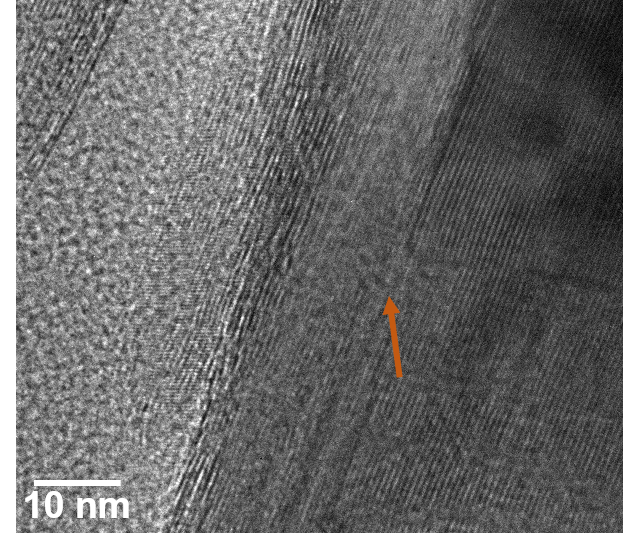
**

**Figure S16.** TEM image of BN/S/C where we speculate that S species attached on the surface (marked with arrow) of BN is observed.

**
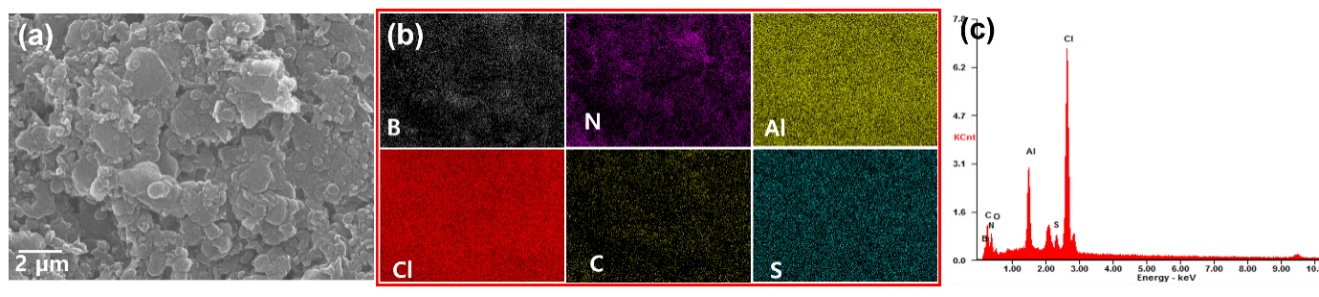
**

**Figure S17.** (**a**) SEM image and (**b**,**c**) EDX mapping of BN/S/C electrode charged to 2.2 V vs. AlCl_4_^-^/Al.

**
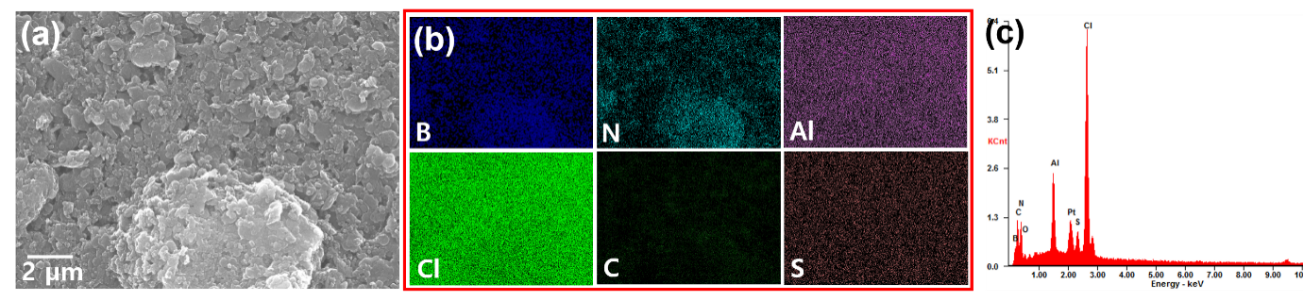
**

**Figure S18.** (**a**) SEM image and (**b**,**c**) EDX mapping of BN/S/C electrode discharged to 0.05 V vs. AlCl_4_^-^/Al.

**
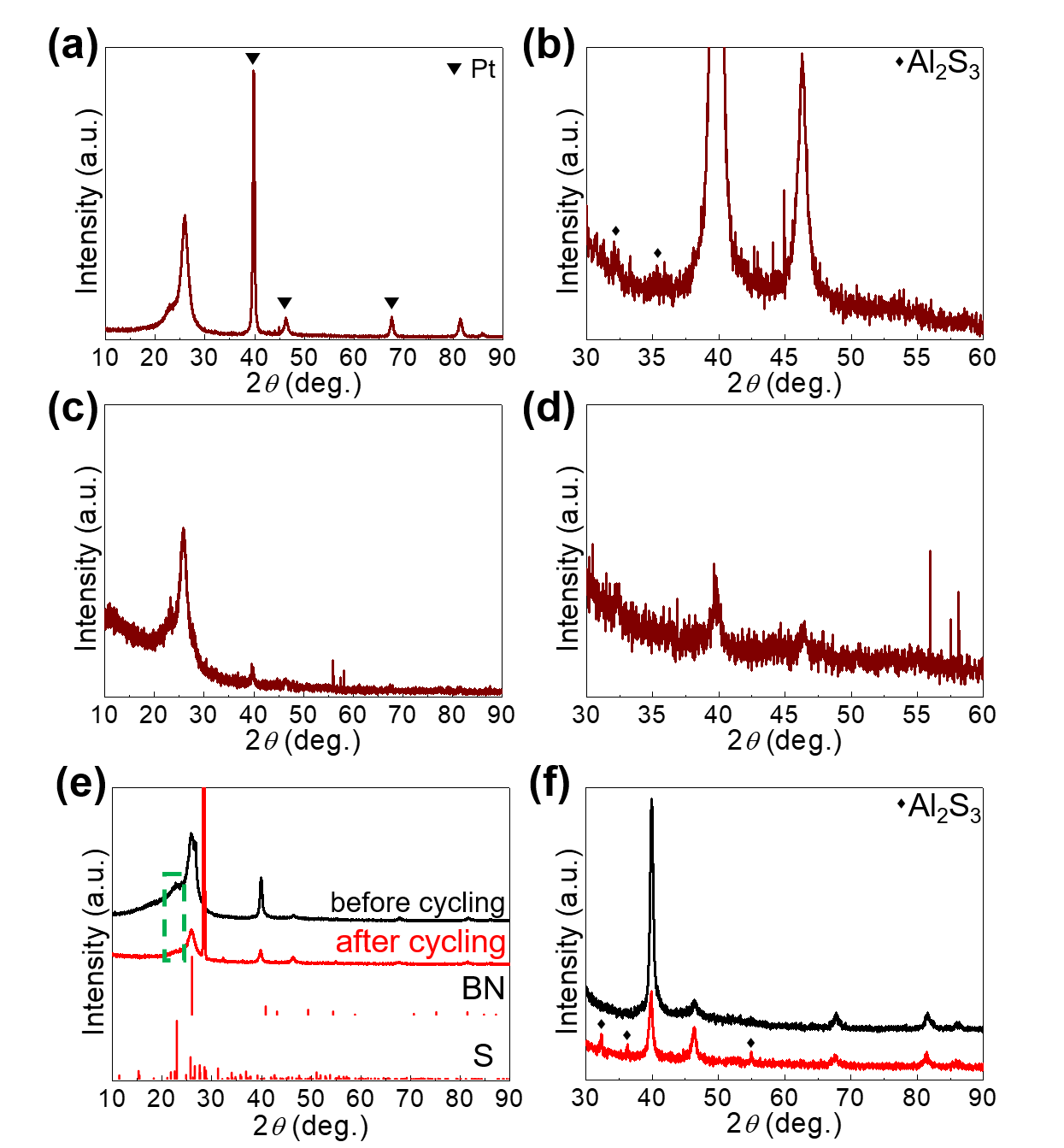
**

**Figure S19.** XRD spectra of BN/S/C discharged to (**a**,**b**) 0.05 V vs. AlCl_4_^-^/Al and (**c**,**d**) 0.7 V vs. AlCl_4_^-^/Al. (**e**) XRD spectra of BN/S/C before and after the repeated charge/discharge cycling measurement. S peak in the fresh electrode disappears after charge/discharge cycling test, while layered BN is well preserved. (**f**) Magnified XRD spectra of BN/S/C where the formed Al_2_S_3_ phase during discharge process is detected.

**
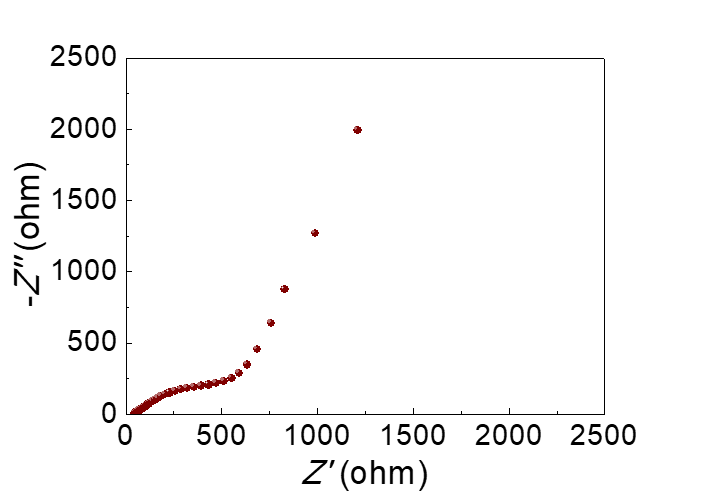
**

**Figure S20.** EIS spectra of the Al//[EMIM]Cl·AlCl_3_//BN/S/C cell before repeated charge/discharge cycling tests.

**Table S1.** Systematic comparison of electrochemical performances (including synthesis method, discharge plateau, capacity, and cycling number) of the reported cathode materials for AIBs, ASBs, and Al dual-ion batteries.

| **Material** | **Synthesis**  **method** | **Discharge plateau (V)** | **Current density**  **(A g^-1^)** | **Capacity**  **(mAh g^-1^)** | **Cycling number** | **Ref.** |
| --- | --- | --- | --- | --- | --- | --- |
| S powder | Mixed S/C | ~1.2 | 0.05 | ~1,300 | 6 | 1 |
| S/activated carbon cloth | Melt-diffusion | ~0.5 | 0.05 | ~1,000 | 20 | 2 |
| S/mesoporous carbon | Melt-diffusion | ~0.5 | 0.251 | ~400 | 20 | 3 |
| S/carbon nanofiber | Dispersing loading | ~0.76 | 0.021 | ~600 | 50 | 4 |
| 3D graphene foam | CVD & wet-chemical etching | ~1 | 2 | ~23 | 250 | 5 |
| Graphite paper | — | 2.3 & 2 | 0.02 | ~70 | 600 | 6 |
| 3D graphitic foams | Electrochemical expansion & heat treatment & hydrogen evolution | 1.8 | 12 | ~60 | 4,000 | 7 |
| Defect-free graphene | High-temperature annealing & mechanical compression | 2.3 & 1.8 | 5 | ~100 | 25,000 | 8 |
| Graphite | — | ~4 | 0.2 | ~90 | 200 | 9 |
| Graphite | — | ~1.7 | 0.5 | ~60 | 1,000 | 10 |
| Porous graphite | High-temperature polarization | ~1.7 | 10 | 104 | 3,000 | 11 |
| Graphite | — | ~2.2 & 1.7 | 0.198 | ~110 | 6,000 | 12 |
| Pyrolytic graphite | CVD growth & wet-chemical etching & annealing | 2.25 & 1.7 | 4 | ~60 | 7,500 | 13 |
| Carbon nanoscrolls | Carbonization of soft carbon & wet-chemical etching | 1.45 | 0.1 | ~100 | 6,600 | 14 |
| Graphene nanoribbons | CVD & heat-treatment & plasma and wet-chemical etching | ~1.65 | 5 | 123 | 10,000 | 15 |
| C@N-C@N,P-C | Three-step wet chemical growth & pyrolysis & wet chemical etching | — | 5 | 98 | 2,500 | 16 |
| Large-sized few-layer graphene | Solution filtration | 2.25 & 1.78 | 0.3 | 76.5 | 7,000 | 17 |
| Zeolite-templated carbon | Two-step method | — | 0.1 | 178.1 | 500 | 18 |
| Co_3_S_4_ microsphere | Solve-thermal | ~0.68 | 0.05 | ~90 | 150 | 19 |
| CoSe nanoparticles | Hydrothermal & heat-treatment | 1.9 & 1 | 5 | 60 | 100 | 20 |
| Cobalt sulfde@carbon  nanotube | Electrospinning & calcination & CNT growth & sulfidization | ~0.95 | 0.1 | 297 | 200 | 21 |
| Ni_3_S_2_@ graphene | Grinding & heat-treatment | ~1 | 0.1 | 60 | 100 | 22 |
| Graphene-modified VS_2_ | Wet-mixing | ~0.6 | 0.1 | 50 | 50 | 23 |
| V_2_O_5_ | Wet-chemical/heat-treatment | ~0.6 | 0.044 | ~180 | 5 | 24 |
| SnS_2_/rGO | Heating/refluxing & heat-treatment & atmosphere annealing | 0.68 | 0.2 | 70 | 100 | 25 |
| VS_4_/rGO | Hydrothermal | — | 0.1 | 80 | 100 | 26 |
| CuS@C | Solvothermal treatment | ~1.0 | 0.02 | 90 | 100 | 27 |
| Cu_2−x_Se nanorods | Water evaporation process | 0.5~ | 0.2 | 100 | 100 | 28 |
| ordered WO_3-x_ nanorods | Hydrothermal | ~1.4 & 1 | 0.1 | ~60 | 100 | 29 |
| Porous microspheric copper oxide | Hydrothermal | 0.6 | 0.2 | ~112 | 100 | 30 |
| MoS_2_ microsphere | Hydrothermal | — | 0.04 | 66.7 | 100 | 31 |
| CoSe_2_/carbon nanodice@rGO | Wet-chemical & selenization & GO wrapping & annealing | ~1.9 & 0.9 | 1 | 143 | 500 | 32 |
| Vanadium carbide | Ball-milling & sintering & wet-chemical etching | ~1 | 0.1 | 90 | 100 | 33 |
| BN/S/C | Ball-milling | ~1.15 | 0.1 | 532 | 300 | This work |

**Note:**

1. Capacities recorded in this table are the remaining capacity values at the end of repeated charge/discharge cycling tests.

2. Ref. no.1 to no.4 are Al-S battery. Others are general AIBs or Al dual-ion batteries.

3. Voltage plateau is determined according to the values in the middle of the discharge plateaus.

4. Synthesis process excluding slurry casting mainly refers to the preparation of original active materials.

5. “—” Represents an unavailability of cathode materials for relevant properties.

**References**

1. Cohn, G., Ma, L. & Archer, L. A. A novel non-aqueous aluminum sulfur battery. *J. Power Sources* **283**, 416–422 (2015).

2. Gao, T. *et al*. A rechargeable Al/S battery with an ionic‐liquid electrolyte. *Angew. Chem. Int. Edit.* **55**, 9898–9901 (2016).

3. Yang, H. *et al*. An aluminum-sulfur battery with a fast kinetic response. *Angew. Chem. Int. Edit.* **57**, 1898–1902 (2018).

4. Yu, X., Boyer, M. J., Hwang, G. S. & Manthiram, A. Room-temperature aluminum-sulfur batteries with a lithium-ion-mediated ionic liquid electrolyte. *Chem-US* **4**, 586–598 (2018).

5. Zhang, E. *et al*. A novel aluminum dual-ion battery. *Energy Storage Mater.* **11**, 91–99 (2018).

6. Wang, S. *et al*. A novel dual-graphite aluminum-ion battery. *Energy Storage Mater.* **12**, 119–127 (2018).

7. Wu, Y. *et al*. 3D graphitic foams derived from chloroaluminate anion intercalation for ultrafast aluminum‐ion battery. *Adv. Mater.* **28**, 9218–9222 (2016).

8. Chen, H. *et al.* A defect‐free principle for advanced graphene cathode of aluminum‐ion battery. *Adv. Mater.* **29**, 1605958 (2017).

9. Zhang, X., Tang, Y., Zhang, F. & Lee, C.‐S. A novel aluminum-graphite dual‐ion battery. *Adv. Energy Mater.* **6**, 1502588 (2016).

10. Li, Z., Liu, J., Niu, B., Li, J. & Kang, F. A novel graphite-graphite dual ion battery using an AlCl_3_-[EMIm]Cl liquid electrolyte. *Small* **14**, 1800745 (2018).

11. Zhang, C. *et al*. Amorphous carbon-derived nanosheet-bricked porous graphite as high-performance cathode for aluminum-ion batteries. *ACS Appl. Mater. Inter.* **10**, 26510–26516 (2018).

12. Wang, D. Y. *et al*. Advanced rechargeable aluminum ion battery with a high-quality natural graphite cathode. *Nat. Commun.* **8**, 14283; 10.1038/ncomms14283 (2017).

13. Lin, M.-C. *et al*. An ultrafast rechargeable aluminium-ion battery. *Nature* **520**, 324 (2015).

14. Liu, Z. *et al*. Carbon nanoscrolls for aluminum battery. *ACS Nano* **12**, 8456–8466 (2018).

15. Yu, X., Wang, B., Gong, D., Xu, Z. & Lu, B. Graphene nanoribbons on highly porous 3D graphene for high‐capacity and ultrastable Al‐ion batteries. *Adv. Mater.* **29**, 1604118 (2017).

16. Li, C. *et al*. Heteroatomic interface engineering in MOF-derived carbon heterostructures with built-in electric-field effects for high performance Al-ion batteries. *Energy Environ. Sci.* **11**, 3201–3211 (2018).

17. Zhang, L., Chen, L., Luo, H., Zhou, X. & Liu, Z. Large‐sized few‐layer graphene enables an ultrafast and long‐life aluminum‐ion battery. *Adv. Energy Mater.* **7**, 1700034 (2017).

18. Stadie, N. P., Wang, S., Kravchyk, K. V. & Kovalenko, M. V. Zeolite-templated carbon as an ordered microporous electrode for aluminum batteries. *ACS Nano* **11**, 1911–1919 (2017).

19. Li, H. *et al*. A highly reversible Co_3_S_4_ microsphere cathode material for aluminum-ion batteries. *Nano Energy* **56**, 100–108 (2019).

20. Xing, W. *et al*. Carbon-encapsulated CoSe nanoparticles derived from metal-organic frameworks as advanced cathode material for Al-ion battery. *J. Power Sources* **401**, 6–12 (2018).

21. Hu, Y. *et al*. A binder‐free and free‐standing cobalt sulfide@carbon nanotube cathode material for aluminum‐ion batteries. *Adv. Mater.* **30**, 1703824 (2018).

22. Wang, S. *et al*. A novel aluminum‐ion battery: Al/AlCl_3_‐[EMIm]Cl/Ni_3_S_2_@graphene. *Adv. Energy Mater.* **6**, 1600137 (2016).

23. Wu, L. *et al*. A rechargeable aluminum-ion battery based on a VS_2_ nanosheet cathode. *Phys. Chem. Chem. Phys.* **20**, 22563–22568 (2018).

24. Wang, H. *et al*. Binder-free V_2_O_5_ cathode for greener rechargeable aluminum battery. *ACS Appl. Mater. Inter.* **7**, 80–84 (2014).

25. Hu, Y. *et al*. An innovative freeze‐dried reduced graphene oxide supported SnS_2_ cathode active material for aluminum‐ion batteries. *Adv. Mater.* **29**, 1606132 (2017).

26. Zhang, X. *et al*. Flower‐like vanadium suflide/reduced graphene oxide composite: an energy storage material for aluminum‐ion batteries. *Chem. Sus. Chem.* **11**, 709–715 (2018).

27. Wang, S. *et al*. High-performance aluminum-ion battery with CuS@C microsphere composite cathode. *ACS Nano* **11**, 469–477 (2016).

28. Jiang, J. *et al*. One-dimensional Cu_2-x_Se nanorods as the cathode material for high-performance aluminum-ion battery. *ACS Appl. Mater. Inter.* **10**, 17942–17949 (2018).

29. Tu, J., Lei, H., Yu, Z. & Jiao, S. Ordered WO_3-x_ nanorods: facile synthesis and their electrochemical properties for aluminum-ion batteries. *Chem. Commun.* **54**, 1343–1346 (2018).

30. Zhang, X., Zhang, G., Wang, S., Li, S. & Jiao, S. Porous CuO microsphere architectures as high-performance cathode materials for aluminum-ion batteries. *J. Mater. Chem. A* **6**, 3084–3090 (2018).

31. Li, Z., Niu, B., Liu, J., Li, J. & Kang, F. Rechargeable aluminum-ion battery based on MoS_2_ microsphere cathode. *ACS Appl. Mater. Inter.* **10**, 9451–9459 (2018).

32. Cai, T. *et al.* Stable CoSe_2_/carbon nanodice@reduced graphene oxide composites for high-performance rechargeable aluminum-ion batteries. *Energy Environ. Sci.* **11**, 2341–2347 (2018).

33. Vahidmohammadi, A., Hadjikhani, A., Shahbazmohamadi, S. & Beidaghi, M. Two-dimensional vanadium carbide (MXene) as a high-capacity cathode material for rechargeable aluminum batteries. *ACS Nano* **11**, 11135–11144 (2017).
